# Therapeutic Approaches Targeting Nucleolus in Cancer

**DOI:** 10.3390/cells8091090

**Published:** 2019-09-16

**Authors:** Pietro Carotenuto, Annalisa Pecoraro, Gaetano Palma, Giulia Russo, Annapina Russo

**Affiliations:** 1 The Institute of Cancer Research, Cancer Therapeutic Unit, London SM2 5NG, UK; carotenuto.pietro@yahoo.com; 2 Telethon Institute of Genetics and Medicine (TIGEM), Pozzuoli 80078, Italy; 3 Department of Pharmacy, School of Medicine, University of Naples Federico II, Via Domenico Montesano 49, 80131 Naples, Italy; annalisa.pecoraro@unina.it; 4 Department of Advanced Biomedical Science, School of Medicine, University of Naples Federico II, 80131 Naples, Italy; gaetano.palma@unina.it

**Keywords:** nucleolar stress, p53, ribosomal proteins, cancer, uL3, cancer chemotherapy, nucleolus

## Abstract

The nucleolus is a distinct sub-cellular compartment structure in the nucleus. First observed more than 200 years ago, the nucleolus is detectable by microscopy in eukaryotic cells and visible during the interphase as a sub-nuclear structure immersed in the nucleoplasm, from which it is not separated from any membrane. A huge number of studies, spanning over a century, have identified ribosome biogenesis as the main function of the nucleolus. Recently, novel functions, independent from ribosome biogenesis, have been proposed by several proteomic, genomic, and functional studies. Several works have confirmed the non-canonical role for nucleoli in regulating important cellular processes including genome stability, cell-cycle control, the cellular senescence, stress responses, and biogenesis of ribonucleoprotein particles (RNPs). Many authors have shown that both canonical and non-canonical functions of the nucleolus are associated with several cancer-related processes. The association between the nucleolus and cancer, first proposed by cytological and histopathological studies showing that the number and shape of nucleoli are commonly altered in almost any type of cancer, has been confirmed at the molecular level by several authors who demonstrated that numerous mechanisms occurring in the nucleolus are altered in tumors. Recently, therapeutic approaches targeting the nucleolus in cancer have started to be considered as an emerging “hallmark” of cancer and several therapeutic interventions have been developed. This review proposes an up-to-date overview of available strategies targeting the nucleolus, focusing on novel targeted therapeutic approaches. Finally, a target-based classification of currently available treatment will be proposed.

## 1. Introduction

The nucleolus is a well-characterized sub-nuclear structure, visible by microscopy inside the nucleoplasm as a distinct compartment not separated by any membrane. In late mitosis, nucleoli are assembled around tandem clusters of ribosomal genes (rDNA) called “nucleolar organizer regions” (NORs) [1,2]. During the interphase, the nucleolus acquires a dynamic structure to accommodate its canonical molecular function: the biogenesis of ribosomes. The ribosome biogenesis is a multi-step process, functionally organized to take place in three sub-nucleolar compartments: the fibrillar compartment (FC), the dense-fibrillar compartment (DFC), and the granular compartment (GC). Ribosome biogenesis is an energy-consuming and well-orchestrated process, in which all the constituents of the ribosomes are synthetized, modified, assembled in the nucleolus, and finally carried into the cytoplasm to build up the mature ribosomes [3]. Besides the constituents of the ribosomes, namely ribosomal rRNAs and ribosomal proteins (RPs), a large number of molecular players are involved in ribosome biogenesis, such as RNA polymerases (RNA Pol), small nucleolar RNAs (snoRNAs), regulatory, processing, assembling, and maturation factors [3]. Ribosome biogenesis starts in the FC, where the transcription of rDNA genes by the RNA Pol I results in the synthesis of the rRNA precursor 47S. In the nucleus, RNA Polymerase III synthesizes 5S rRNA that will be subsequently accumulated in the nucleolus. Belonging to the transcription machinery, several factors such as Topoisomerase I (Top I), Upstream Binding Factor (UBF), the transcription initiation factor RRN3, and the selectivity factor SL1, play a key role in the biosynthesis of the 47S rRNA precursor [2]. The processing of the 47S precursor occurs in the DFC [4], an area surrounding the FC, to be further completed in the GC, where the mature rRNAs and RPs are assembled to build up the ribosomal subunits (40S and 60S), ready to be transferred to the cytoplasm to form the mature ribosomes [5,6,7]. Given the fact that the protein biosynthesis is directly coupled with cell growth and proliferation, and dependent on ribosome biogenesis, it is not surprising that ribosome biogenesis plays a crucial role for the orchestration of major cellular processes.

Recent advances have highlighted a large series of non-canonical functions assigned to the nucleolus, independently from ribosome biogenesis. Multiple genomic and proteomic studies [8] have characterized the non-canonical role of the nucleolus in regulating a large number of the major cellular processes including the maintenance, repair and stability of the genome [2,9,10], the cell-cycle [11], cellular senescence [12], response to stress [13,14,15], telomere maintenance [16], and the nuclear architecture [2].

Several human diseases have been associated with nucleolar dysfunction. Mutations in genes encoding ribosome components or ribosome biogenesis factors were identified by several authors and linked to a class of human inherited disorders called “Ribosomopathies” [11]. The nucleolus has also been linked with numerous viral infection and nucleolar activities showed to be essential for virus replication and/or pathogenesis [11].

Furthermore, alterations in both canonical and non-canonical functions of the nucleolus have been associated with several forms of cancer [8,17,18,19,20]. In this review, the association between nucleolus and cancer will be summarized, and the “druggability” of nucleolus extensively discussed. An overview of available strategies targeting the nucleolus focusing on the novel targeted therapeutic approaches will be presented and a target-based classification of currently available treatment will be proposed.

## 2. Nucleolus and Cancer 

The functional link between the nucleolus and cancer has been assessed by several cytological and histopathological studies. Almost all cancer types display abnormalities in their morphology and number of nucleoli [21,22]. In some cancers, nucleolar size has been used as predictive and prognostic biomarker in chemotherapeutic treatment [21] and clinical outcomes [22]. 

Numerous evidences collected over decades has demonstrated that the abnormalities in the morphology and numbers of nucleoli are the direct consequence of the over-activation of ribosome biogenesis in cancer [23]. The over-activation of ribosome biogenesis is directly dependent on the abnormal need of tumor cells to produce proteins sustaining their altered growth. Several findings showed that the molecular players taking part in the ribosome biosynthetic core-machine are over-activated in cancer rather than in normal cells, thus leading several authors to propose that cancer cells are “addicted” to the over-activation of ribosome biogenesis [20,24,25]. The ribosome biogenesis “addiction” is an important concept in cancer therapeutic, especially for the development of targeted-based therapies able to target specifically the above described cancer-specific molecular alteration [26,27,28]. Many authors have shown that in cancer cells, the altered activity of the rRNA transcriptional machinery is mechanistically the cause of ribosome biogenesis over-activation [29]. Importantly, the activity of the nucleolar-resident RNA Pol I, the main actor in rRNA precursor transcription, is frequently elevated in cancer, and RNA Pol I over-activity has been correlated with adverse prognosis in several tumors [30,31]. For example, as reported by Bywater M.J. et al. the over-activation of rDNA transcription, mediated by the increased activity of RNA Pol I in hematologic cancers, is required for the proliferation of tumor cells [32]. Several authors have suggested that the altered activity of the RNA Pol I is not caused by genetic alteration in the RNA Pol I, but mainly by the dysregulation of the major cancer-related signalling pathways like Myc, RAS/RAF/ERK, PI3K/AKT/mTOR, p53, pRb and PTEN [20,29,33,34]. In fact, a large array of information from cancer-related pathways converge on the nucleolus to regulate the ribosome production that, in turn, drives cancer growth and proliferation. The role of the cancer-related pathways in the regulation of nucleolar functions will be addressed in the following paragraphs.

However, recently, results from different groups have led to the proposal of a new concept, that of a pluri-functional nucleolus, providing evidence that the nucleolus exerts non-canonical functions independently from ribosome biogenesis [1,13,20]. The non-canonical role of the nucleolus in regulating a large number of cellular processes such as the maintenance, repair, and stability of the genome, the cell-cycle, cellular senescence, response to the stress, telomere regulation, and the nuclear architecture has been exploited by several authors and associated to cancer [2,9,10,11,12,13,14,15,16].

Furthermore, the expression of several RPs has been found to be altered in human tumors such as colorectal cancer, esophagus cancer, and hepatocellular carcinoma [13,14]. Further, the genetic alteration of genes encoding for RPs has also been detected in cancer samples, suggesting their role as oncogenes or tumor suppressors. For example, mutations in uL5 are observed in melanoma and T-cell acute lymphoblastic leukemia, and deletions or inactivating mutations of uL18 occurs in T-cell acute lymphoblastic leukemia and in multiple myeloma, melanoma, glioblastoma and breast cancers [35,36].

Recently, investigations mapping the nucleolar proteome have proposed that RPs and nucleolar proteins are involved in cancer-related cellular functions by sequestering in the nucleolus tumor associated key proteins [8,37].

Several oncogenes and tumor suppressor gene were found to be regulated by the RP-mediated nucleolar sequestering such as p53, Cdc14B, PICT1, Cyclin D1, ErbB3, GNL1, BCL-2, PCNA, RAD51, c-myc and NF-kb [13,38,39,40,41,42]. Nucleolin, the most abundant protein in the nucleolus, was found over-expressed in several tumors [43]. At the molecular level, Nucleolin acts using an RP-sequestering mechanism by interacting with cell cycle-regulators as RPA (replication protein A) and several DNA repair proteins such as PCNA, gamma-H2AX and RAD51, thus facilitating cancer progression [44,45]. Nucleolin exerts also a regulation control against BCL-2 by directly binding the 3′UTR of BCL-2 mRNA and interacting with 15a and 16 miRNAs, negative regulators of BCL-2 expression [46]. The nucleolar protein SCF^Fbw7^, a member of SCF family of ubiquitin E3 ligases, using a similar molecular mechanism, regulates some proteins required for cancer cell proliferation like Cyclin E and MYC [47]. 

**Figure 1:**
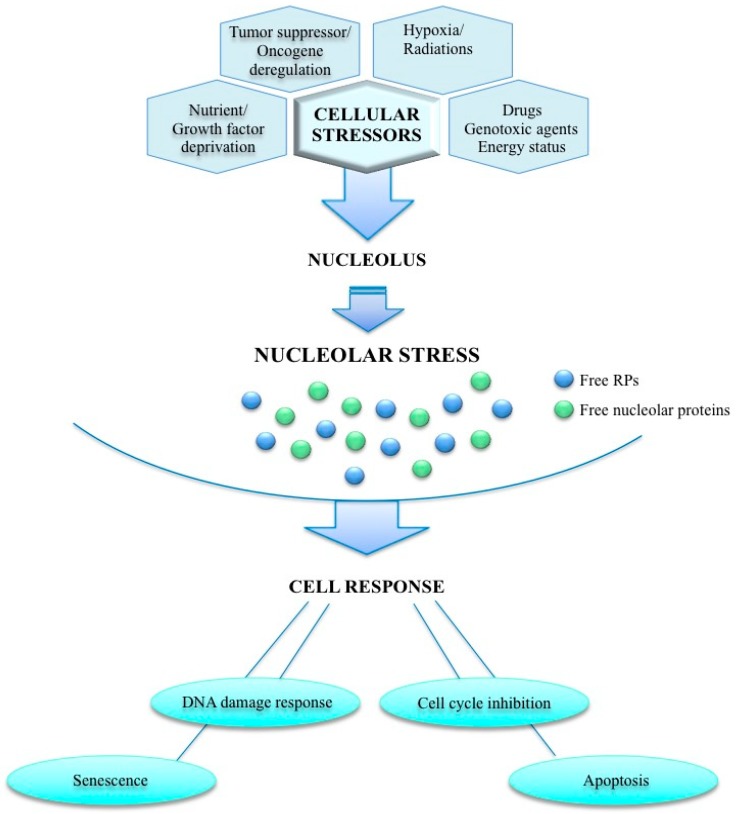
The nucleolar stress pathway. Nucleolus can be exposed to a variety of cellular stressors that disrupt ribosome biogenesis activating a complex cellular response namely “nucleolar stress”. This stress pathway is mediated by several ribosomal proteins RPs and/or nucleolar proteins and its activation results in cell cycle arrest, apoptosis, DNA damage and senescence. Dysregulation of this response is known to contribute to the development of cancer.

A wide range of stress stimuli (radiations, oncogenes, nutrient deprivation, hypoxia, genotoxic compounds) may disrupt ribosome biogenesis and activate a complex cellular response, namely nucleolar stress. The nucleolar stress pathway activation, mediated by several nucleolar and RPs, results in cell-cycle blocking, activation of apoptosis, DNA damage and senescence. In Figure 1, a schematic model of the nucleolar stress pathway has been proposed. Alteration of the nucleolar stress pathway is known to contribute to the development of cancer [13]. 

The nucleolar stress pathway integrates several networks, some of which require the p53 activity while others are independent [13,48]. 

Several studies have elucidated the role of the p53-binding protein MDM2 (mouse double minute 2) in nucleolar stress pathway. The existence of an RNP-network complex regulating MDM2 activity has been reported [49]. The RNP-MDM2-p53 pathway is activated by stress stimuli resulting in the release of some RPs from the nucleolus to the nucleoplasm. The interaction between RPs and MDM2 results in the p53 stabilization and subsequent blocking of the cell cycle and apoptosis [13,50]. The p53-mediated nucleolar stress pathway will be discussed in details in following paragraphs.

Emerging evidence attests to the existence of several nucleolar stress pathways not dependent on p53 involvement. Several factors participating in p53-independent networks, such as nucleophosmin 1 (NPM1), peter pan homolog (PPAN), and the p14 alternative reading frame protein (p14ARF) [13]. In response to stress signalling stimuli, the above listed nucleolar proteins act by sequestering the activity of several cell cycle regulatory proteins and inhibit cell proliferation [34]. Besides from taking part to ribosomal biogenesis, NPM1 is a multifunctional protein over-expressed in many types tumors including tumors of colon, liver, stomach, ovary and prostate [51]. Recently, PPAN, an NPM1 interactor, was found to be over-expressed in embryonal and intestinal cancers constitutively active for Wnt signalling [52]. Several lines of evidence showed that p14ARF is a tumor suppressor gene and the regulator of a wide range of molecular partners—p14ARF activation is triggered by oncogenic and genotoxic stresses, resulting in DNA damage pathways and cell-cycle inhibition [40,53,54]. 

A number of studies conducted by our group have brought to light a key role of the RP uL3 in the p53-independent nucleolar stress pathway [13,55,56]. Besides being a component of a large ribosomal subunit, uL3 is also a member of a subset of RPs that, as free proteins, are directly implicated in various extra-ribosomal functions that require specific mechanisms of regulation [57,58,59]. Further, uL3 is able to auto-regulate its own expression in combination with a complex protein network to including heterogeneous nuclear ribonucleoprotein H1 (hnRNPH1), KH-Type splicing regulatory protein (KHSRP) and NPM1 [60,61,62]. Free uL3 is also involved in selective gene regulation via cystathionine-*β*-synthase (CBS) [63] and p21 pathways [64,65]. Moreover, uL3 acts as regulator of cancer-related signaling pathways such as NFkB pathway and ERK [66,67]. The active ERK is essential in mediating uL3-induced p21 expression [68]. More recently, it has been shown that uL3 is a mediator of nucleolar stress induced by several chemotherapeutic drugs as 5-FU, oxaliplatin, Actinomycin D and Niclosamide in p53-mutated lung and colon cancer cells [56,68,69,70]. In particular, by regulating the levels of p21 and CBS proteins, the uL3 protein is able to sensitize the resistant cells to chemotherapeutic compounds, strongly suggesting a key role in drug-response in cancer [71].

The above described findings on uL3, and several other studies mapping the nucleolar proteome, have demonstrated the role of the nucleolus in integrating several stress signalling pathways impaired in cancer cells [13,37]. 

The nucleolar proteome and its correlation with cancer-related processes has been largely discussed above, but a great deal of attention are receiving the nucleolar non coding RNAs, such as the small nucleolar RNA (snoRNAs) and the rDNA-hosted pre-miRNA analogs (rmiRNAs) recently identified as regulators of nucleolar stress or of cancer-related genes [72,73]. In the same way, the long non-coding RNAs (lncRNAs), another emerging class of non-coding RNAs frequently de-regulated in cancer [74], are involved in the regulation of several both canonical and non-canonical nucleolar functions [75].

Finally, several evidences have shown the association between rDNA stability and tumorigenesis in human cancer [9]. It is known that rDNA is an extremely variable region of the genome concerning the copy number, and tumor cells have lower rDNA copy number than normal tissue. The loss of copy of rDNA is associated with the sensitivity to drugs acting as DNA-damaging agents, thus the rDNA copy number can be used as predictive biomarker in chemotherapy [76].

In summary, several functions have been attributed to the nucleolus. Numerous recently identified non-canonical functions have been added to the canonical and common accepted function of the site and regulator of the biogenesis of ribosomes. In the last years, several hallmark genomic and proteomic works have provided fascinating insights into the molecular associations between nucleolus and cancer. It has been showed that during tumor progression, a series of oncogenic stimuli converge at the nucleolus triggering the over-activation of the ribosome biogenesis and/or by producing alteration of nucleolar stress pathways. A growing understanding of the molecular association between nucleolus and cancer is driving the development of a new generation of anticancer drugs, specifically targeting the molecular nucleolar-related functions underlying tumor formation and progression.

## 3. Targeting Nucleolus in Cancer

Several therapeutic approaches have been developed to target nucleolus and their relevance in cancer therapeutic has been largely confirmed [20,77]. In the subsequent sections we will provide an update on recent treatments and propose a general classification of current available compounds (Table 1 and Table 2).

**Figure 2:**
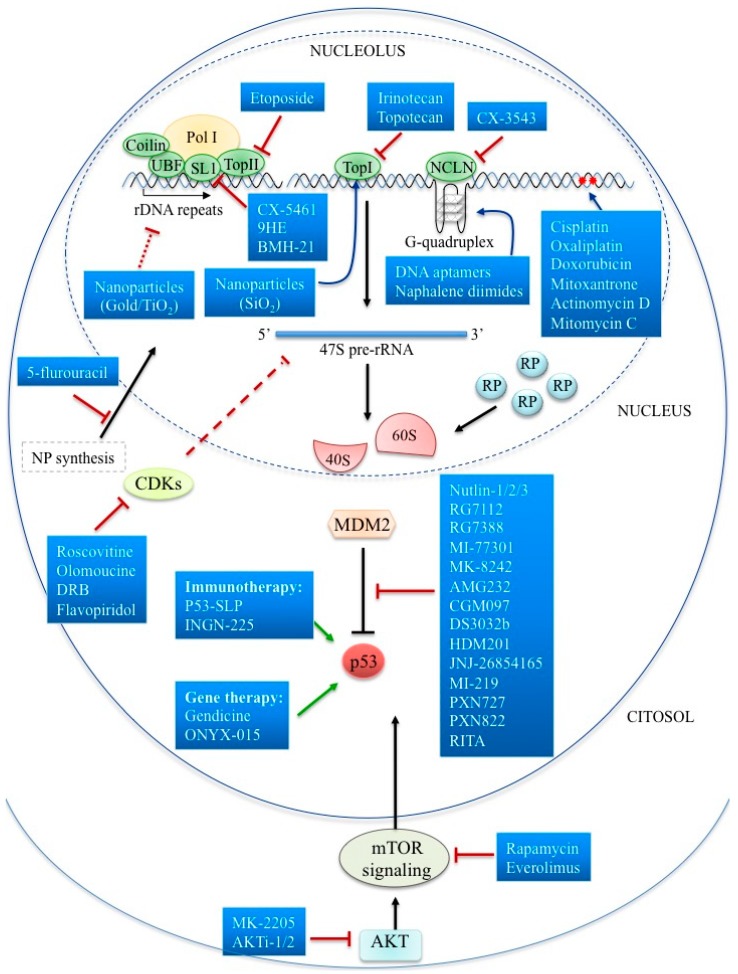
Targeting Nucleolar Function in Cancer Therapeutics. Schematic representation of anti-cancer drugs targeting nucleolar structures and/or functions.

The next section presents a functional classification of drugs targeting the nucleolus in cancer. The first class of compounds includes several molecules targeting directly or indirectly the nucleolus structures or functions. To this set of first-generation compounds belong agents that act mainly by disrupting nucleolar integrity, such as rDNA intercalating, alkylating crosslinking agents, or interfering with rDNA transcription and maturation. The second and third classes of nucleolar targeting agents are composed of the inhibitors of ribosome biogenesis, to which belong RNA Pol I direct inhibitors and compounds targeting molecular signalling pathways regulating ribosome biogenesis. Chemotherapeutics and small compounds activating the p53-mediated nucleolar stress pathway constitute another therapeutic approach. Recently, nucleolar hub proteins acting by the nucleolar-sequestering mechanism have been suggested as molecular targets in therapeutic interventions. Recent works depicting the nucleolar proteome and interactome map were crucial in driving the development of new drugs targeting the nucleolus. Furthermore, the elucidation of nucleolus cancer-related functions specifically altered during cancer development will benefit the growing area of personalized medicine with new targeting approaches. This review will discuss old and novel therapeutic strategies targeting the nucleolus in cancer, focusing in particular on novel targeted approaches as a promising class of drugs for cancer therapy. In Figure 2, a comprehensive schematic representation of currently targeting approaches is reported.

## 4. Targeting the Nucleolar Components

DNA-alkylating agents, nucleotide analogues, and anthracyclines constitute a large class of DNA-damaging drugs, already approved for clinical use in cancer, known to exert their anticancer activity by blocking DNA replication with the consequent induction of apoptosis [78]. In particular, nitrogen mustard and its derivatives alkylate DNA on purine bases, and some of them can form inter-strand cross links on DNA. Alkylating-like platinum drugs, i.e., cisplatin and its platinum-based analogs form intra-strand cross links on DNA that are associated to apoptosis if not repaired. Nucleotide analogues such as the pyrimidine analog 5-fluorouracil (5-FU), and the purine analogs 6-mercaptopurine and 8-azaguanine are incorporated into DNA during the S phase of the cell cycle with the consequent block of DNA duplication and cell death. Anthracyclines act by blocking the interaction between DNA and Topoisomerase II and are also able to intercalate between bases [78]. Besides that, it is now clear that most of these drugs are also able to target the main nucleolar components causing the disruption of nucleolar structures. The following discussion will describe the above-mentioned compounds in light of their activity on targeting nucleolar components and related structures (nucleolar-resident rDNA, rRNA, RPs) (Table 1). Considering these activities, DNA-alkylating agents, nucleotide analogues and anthracyclines, can be classified as rRNA transcription inhibitors. Drugs such as cisplatin and oxaliplatin are able to inhibit RNA Polymerase I by forming platinum adducts with rDNA, thus creating a steric impediment to polymerase activity. It has been shown that platinum adducts are also able to bind some nucleolar proteins, including DNA repair factors, the transcription factors UBF, and the Poly ADP-ribose polymerase 1 (PARP 1), as well as HMG-domain proteins, thus interfering with their functions [37].

Several antibiotics of the anthracycline group, such as Doxorubicin and Mitoxantrone, acting as DNA intercalators and inhibitors of Topoisomerase II activity, are in use for the treatment of a wide range of cancers, including both haematological and solid tumors [29]. Belonging to the same class, Mitomycin C is able to alkylate guanosines and crosslink rDNA followed by further blocking of the Pol I [29].

Actinomycin D (Act D) is another potent intercalating agent in clinical use for the treatment of Wilm’s tumor and several type of sarcoma. Act D binds to dsDNA especially at the 3′ side of guanine residues, in the dinucleotide site GpC and consequently inhibits transcription. High doses of Act D inhibit the transcription of all RNA species. At lower concentrations, i.e., 5nM, Act D specifically inhibits RNA polymerase I driven transcription activating nucleolar stress [68,79,105].

Camptothecin and analogues (Irinotecan and Topotecan), and Epipodophyllotoxins (Etoposide) are potent disruptors of nucleolar integrity by inhibiting Topoisomerase I and II, and consequently resulting in the blocking of Pol I transcription or rRNA processing [79]. 

The basic chemotherapy compound 5-fluorouracil (5-FU), which is widely used in several first-line clinical approved protocols, acts by inhibiting an enzyme involved in nucleotide synthesis, the thymidylate synthase (TS), thus impairing both rDNA and rRNA synthesis. Metabolites of 5-FU act also as rRNA intercalators causing RNA damage which, in turn, leads to nucleolar stress [37,79,80].

Among compounds affecting the nucleolar integrity, there are the Cdk2 (Cyclin-dependent kinase 2) and Cdk9 (Cyclin-dependent kinase 9) inhibitors. The Cdk2 inhibitors Roscovitine and Olomoucine disrupt nucleolar integrity by causing the mis-localization of unprocessed rRNAs and rRNA processing factors [81]. In the same way, the Cdk9 inhibitors DRB (5,6-dichlorobenzimidazone-1-β-d-ribofurano-side) and Flavopiridol promote nucleolar disintegration by inhibiting early rRNA processing and transcription [83]. The alkaloid Flavopereirine (PB-100) has been reported to accumulate in the nucleoli of cancer cells with a potent and specific anti-proliferative activity in several cell lines [82]. 

Finally, data from the literature have demonstrated that proteasome activity and nucleolus organization are linked to each other. Some proteasome inhibitors proposed as drugs or undergoing clinical trials have been reported to target nucleolar morphology and/or function. MG132, a proteasome inhibitor belonging to the class of synthetic peptide aldehydes, induces drastic changes in the localization of the nucleolar markers as NPM for granular component, fibrillarin for dense fibrillar component and UBF for fibrillar center [106].

Ultrastructural analysis demonstrated that Bortezomib, a Food and Drug Administration (FDA)-approved PI in clinical use in mantle cell lymphoma and multiple myeloma [107], causes morphological alterations of the nucleolar ultrastructure associated to the enrichment of the transcription factor ATF4 at nucleolar sites [108].

Recent literature suggests that some nanoparticles (NPs) can provide a useful strategy for the direct delivery of drugs to cancer cells [69,71,109,110]. In particular, some of these NPs are able to specifically target the nucleolus of various cancer cells. SiO_2_-, TiO_2_- and Gold-NPs are the most extensively NPs studied to target the nucleolus in cancer cells. It has been shown that SiO_2_-NPs are able to induce nuclear protein aggregates, in particular Top I aggregates, causing the inhibition of transcription, and consequently inhibiting cancer cell proliferation [83]. Moreover, recent works showed that TiO_2_ nanoparticles conjugated with oligonucleotides specifically matching with rDNA sequences are able to accumulate in the nucleolus depleting it of rDNA, thus providing evidences that nano-drug delivery system constitutes a promising method for a nucleolar-based anti-cancer therapy [84]. Recently, Gold-NPs have been shown to disrupt the nucleolar integrity in breast cancer cell lines by affecting the nucleolar/nucleoplasmatic distribution of several proteins such as the NPM1, the RNA Polymerase I Subunit A (RPA194), the Heat Shock Protein hsp70 and O-GlcNAc-modified proteins [85].

Finally, the anticancer activities of many small molecular compounds able to bind G-quadruplex (G4) structures in DNA have been demonstrated [86,111,112,113]. G4 are nucleic acids structures, particularly abundant in the rDNA of cancer cells. Belonging to these new class of rDNA targeting compounds, the DNA aptamers and naphthalene diimides (NDIs) constitute a class of small molecules able to recognize, induce and stabilize G4 structures with high affinity with a significant potency in inhibiting breast and lung carcinoma cells proliferation [87].

## 5. Targeting Nucleolus by Specific Inhibitors of Ribosome Biogenesis

Numerous chemotherapy-based regimens interfering with ribosome biogenesis, as a result of nuclear structure disruption, were described in the previous paragraph. As discussed before, numerous findings showing the over-activation of ribosome biogenesis in cancer have drawn attention to the targeting of this nucleolar function as a promising approach in cancer therapy [3,20,79]. 

In this paragraph, an update on compounds directly targeting the RNA Pol I as main molecular player of ribosomal biogenesis in cancer will be reported (Table 2). The RNA Pol I activity has been showed to be fundamental for cancer cell proliferation, thus suggesting to develop molecular targeted approaches to selectively inhibit its function in cancer.

The small molecule fluoroquinolone derivative CX-3543 (also referred to as quarfloxin) has been the first compound designed by Cylene Pharmaceuticals to selectively target the RNA Pol I activity. The mechanism of action of CX-3543 is based on targeting and disruption of nucleolin/rDNA G-quadruplex complexes resulting in the inhibition of Pol I transcription and in the apoptosis induction in cancer cells [88]. In phase I clinical trials CX-3543 has been shown to be well tolerated and associated with promising efficacy in patients with solid tumors [33].

Developed by Cylene Pharmaceuticals, the small molecule compound CX-5461 belongs to the next generation of Pol I inhibitors. CX-5461 showed to selectively inhibit the RNA Pol I-dependent transcription by preventing the binding between the rDNA promoter and SL-1, the RNA Pol I transcription initiation factor [89]. Tumorigenicity studies in in vitro cancer models showed the high anti-tumor potency of CX-5461 in a large panel of cancer cells [89]. It has also been demonstrated that the anti-tumor efficacy of CX-5461 is selective for cancer cells and this activity has been also confirmed in in vivo cancer models [89]. The group of M.J. Bywater et al. using mouse transgenic models of hematologic tumors, provided evidence that CX-5461 acts by inducing nucleolar stress mediated by the p53 pathway [32]. Recently it has been demonstrated that CX-5461, in addition to inhibiting rRNA transcription, is also able to binds and stabilize G-quadruplex structures present in DNA with dramatic increase in the number of DNA damage foci in cells. The repair of G4 associated DNA damage is dependent on BRCA1/2-mediated HR and PK-mediated DNA non-homologous end joining (NHEJ pathways. Cells with mutations in genes involved in these mechanisms of DNA damage repair have shown a good response to CX-5461 treatment in in vitro drug sensitivity assays. These results suggest that CX-5461 have the ability to treat effectively tumors deficient in HR and NHEJ repair mechanisms [114].

Finally, the results of the ongoing phase I clinical trial evaluating the efficacy and safety of CX-5461 in patients with haematological cancers will provide novel insights into the therapeutic efficacy of CX-5461, as well as on the potential of therapeutic approaches targeting ribosomal biogenesis in cancer [115].

Several works focused on the anti-tumor effects of the group of alkaloids named ellipticine, for a long time known to act as rDNA-target agents as well as ribosome biogenesis inhibitors. Recently, it has been showed that several ellipticine, mostly the derivative 9-Hydroxyellipticine (9HE), work as specific and potent inhibitors of RNA Pol I, by blocking the interaction between the promoter recognition factor SL1 and the rDNA promoter [90]. A large number of phase I and II clinical trials have evaluated ellipticine derivatives for their efficacy in several cancers, but severe adverse side effects limited their further development [29,90].

By performing an high-throughput screening, the group of K. Peltonen et al. identified the small-molecule BMH-21 as selective inhibitor of RNA Pol I [116]. In particular, BMH-21 is able to intercalate into rDNA binding preferentially GC-rich regions, and to reduce RNA Pol I rDNA association. Furthermore, BMH-21 induces the destabilization and the following degradation of the largest subunit of RNA Pol I, RPA194 [91,92,93]. The rapid degradation of RPA194 results in cancer cell death and is not revealed in normal cells [94]. This activity is specific for polymerase I in fact, and BMH-21 does not cause alteration of RNA Pol II mediated transcription. In vitro and genetic data from yeast mutants suggest that BMH-21 treatment is associated to inhibition of RNA Pol I elongation step that active the degradation of RPA194 [94]. Finally, the involvement of BMH-21 in targeting p53-mediated nucleolar stress pathway has been showed in several cancer cell lines [95]. 

## 6. Targeting Cell Signaling Pathways Functionally Regulating Nucleolus in Cancer

The major cancer-related signal transduction pathways modulate nucleolar functions by regulating the molecular players of ribosome biogenesis (Table 2). The oncogenic signalling mediated by Myc, Ras/ERK, mTOR, and Akt/PKB and tumor suppressor pathways mediated by p53, Rb, ARF, and PTEN alterations converge their signals to nucleolus finely driving the activity of RNA Pol I [24]. 

The oncogenic signalling RAS/RAF/ERK and PI3K/AKT/mTOR (mammalian target of rapamycin), regulate several key-players of the Pol I complex such as RRN3, UBF, and SL-1, resulting in the enhancement of rRNA synthesis in cancer [37]. Furthermore, the oncogene MYC, considered a “master regulator” of ribosome biogenesis, also induces the over-activation of rDNA transcription by affecting the RNA Pol I pre-initiation complex (PIC) formation or by up-regulating the expression of the rDNA transcription factors UBF, SL1, TIF-1A (transcription initiation factor 1A), and POLR1B (polymerase I polypeptide B) [24]. The transcriptional activity of RNA Pol I is also activated by the cell cycle regulator pathway CDK-cyclinD/INK4/pRB/E2F by phosphorylation of UBF [117]. As discussed previously, the p53 pathway, commonly accepted as a main controller in several nucleolar functions, is a negative regulator of RNA Pol I activity. P53 is able to directly interfere with the assembly of PIC by binding to SL-1 and preventing the SL-1/UBF association [118]. Moreover, a key regulator of p53, the tumor suppressor p14ARF inhibits the activity of RNA Pol I by directly altering the PIC formation and blocking the import in the nucleolus of TTF-1 (transcription termination factor 1) [53,54]. 

In recent years, several proteomics works have demonstrated that numerous onco-proteins and tumor suppressors modulate the RNA Pol I transcription such as the cell cycle check point kinase ATM (ataxia telangiectasia mutated) [119], the kinase ATR (ataxia telangiectasia and Rad3-related protein) [120], the DNA-dependent protein kinase DNA-PK [121], the casein kinase CK2 [122], the fusion protein AML1-ETO [123], the transcription factor RUNX2 [124] and NPM1 [125], and more recently identified netrin-1 deltaN isoform [126], DEAD-Box Helicase 31 DDX31 [127] and the zinc finger factors ZNF545/ZFP82 [128].

Several approaches targeting the above described nucleolar regulating pathways are in use in cancer therapeutic strategies. As anticipated before, mTOR is a master regulator of ribosomal biogenesis; two independent mechanisms of action of mTOR pathway take place through the action of two molecular players: the eukaryotic translation initiation factor 4E binding protein 1 (4EBP1) and the ribosomal protein S6 kinase 1 (S6K1). 

The specific inhibitor of the mTOR pathway, including Rapamycin and the new generation ‘rapalog’ (rapamycin analogs), have been well-documented to act as suppressor of rDNA transcription [129]. In particular, the rapalog Everolimus, granted of FDA approval, has been introduced in clinical practice for the treatment of breast cancer and renal cell carcinoma. In a *MYC*-driven lymphoma pre-clinical model characterized by enhanced Pol I transcription Everolimus has showed an high potency in inhibiting the tumor growth [96]. 

The oncogenic PI3K-AKT-mTOR pathway plays an essential role in regulating cancer-related processes such as the resistance to cell death, the cell cycle progression, the angiogenesis, or the metabolism. The protein kinase AKT acts upstream mTOR and regulates other downstream targets of mTOR pathway [129], the influence of which on ribosome biogenesis has been discussed above.

Several AKT inhibitors, such as AKTi-1/2 and MK-2206, have been developed and showed a significative anti-tumor efficacy. Experiments in in vitro and in vivo models showed that their activity is mediated by repressing the rDNA transcription and inducing apoptosis [97]. Further approaches targeting other main pathways regulating the nucleolus in cancer, such as p53, will be discussed in the following paragraphs.

## 7. Targeting p53-dependent Nucleolar Stress Pathway

The p53-dependent nucleolar stress pathway is triggered by several stressing stimuli able to alter the integrity of nucleolus and negatively interfere with ribosome biogenesis leading to the accumulation of p53 (TP53) [13,50]. Several therapeutic approaches have been developed to target the p53-dependent nucleolar stress pathway. The therapeutic strategies developed so far act by restore the tumor suppressor function of p53 in cancer (see Table 2 for a classification) [130]. The modulation of the oncoprotein MDM2 is the key downstream event of the p53-dependent nucleolar stress pathway. 

Two distinct mechanisms are engaged by MDM2 to repress p53: the first and common accepted mechanism is based on the ubiquitination activity of MDM2 which directly binds p53 thus resulting in p53 proteasome-mediated degradation; a second mechanism is ubiquitination-independent and relies on the direct interference of MDM2 with the p53 transcription apparatus [50]. It has been estimated that around 50% of human cancers present mutations in p53 and 17% an aberrant MDM2 expression [131].

For these reasons, the MDM2-p53 network has been the focal point of research in both academia and the industry to develop targeted approaches in cancer therapeutics.

A huge number of basic chemotherapy drugs have been shown to induce the p53-mediated nucleolar stress pathway. Basically, their mechanisms of action rely on DNA damage resulting in a genotoxic stress induction, in the further activation of the p53-mediated nucleolar stress pathway that leads to the inhibition of cancer cell proliferation and apoptosis [23]. Several drugs that act by targeting rDNA, such as alkylating agents, platinum-based drugs, and anthracyclines, have been discussed in the previous paragraphs (see Table 1 for a classification) [37]. In any case, this genotoxic approach causes significant complications, not being able to distinguish between tumorigenic and normal cells. 

With the advent of personalized medicine, non-genotoxic therapeutic approaches to re-activate p53 in cancer have been subjected to an intense investigation in the last years [130] and several compounds have been studied both in pre-clinical and in clinical trials [18]. The major therapeutic approaches include: MDM2 antagonists, inhibitors of the MDM2-p53 interaction, MDM2 antisense targeting the MDM2 expression and inhibitors of RNA Pol I [23]. 

Nutlin-1, Nutlin-2, and Nutlin-3, belonging to the Nutlins family, are small molecules, designed to prevent the MDM2-p53 physical interaction, thus further triggering the activation of p53 [98,99].

RG7112, a Nutlin derivative, is the first developed compound activating the p53 response. Several studies showed its anti-tumor efficacy in a huge number of in vitro and in vivo pre-clinical cancer models and in patients with liposarcoma associated to MDM2 amplification. The results of a phase I study of RG7112 in hematologic malignancies showed clinical activity against relapsed and refractory acute myeloid leukemia AML and chronic lymphocytic leukemia, but pointed out that the treatment was associated with a significative toxicity [98,132]. 

The RG7388, named Idasanutlin, is the lead of a second-generation of MDM2 inhibitors. Idasanutlin showed a significant efficacy in inhibiting the tumor growth both in vitro and in vivo. The efficacy and safety of this compound is under investigation in several clinical trials [100,101].

The spirooxindole-based compound MI-77301 is a small-molecule which prevents the MDM2-p53 interaction by mimicking the p53 protein structure responsible of binding to MDM2. In several tumor cell lines, MI-77301 showed a significative efficacy in inhibiting cancer cell growth and inducing p53-dependent apoptosis [101].

Two phase I clinical trials are currently evaluating the efficacy and safety of the MK-8242, an orally bioavailable, small-molecule inhibitor of the MDM2-p53 interaction. The first study is enrolling patients with solid tumors while the second AML patients to which MK-8242 will be administered in mono-therapy or in combination with cytarabine. In vitro cancer models have previously demonstrated the potency of this compound in inhibiting cancer cell growth with an IC_50_ value of 20 nM [101]. 

The piperidinone-based molecular compound AMG232, able to selectively inhibit MDM2-p53 interaction, has shown a significative anti-tumor efficacy in a large panel of tumor cell lines as well as in mouse models [102]. 

Among the compounds mimicking the p53 peptide structures and interfering with MDM2 interaction sites, a structure-based design has also identified the CGM097 compound as a potent MDM2 inhibitor and activator of p53. The CGM097 is a dihydroisoquinolinone derivative under investigation in a phase I clinical trial evaluating the efficacy of CGM097 in p53 wild-type patients with advanced refractory solid tumors [103]. 

Three clinical studies are currently evaluating the safety, the tolerability and the pharmacokinetics of the novel potent MDM2 inhibitor DS3032b, also known as Milademetan, in patients with AML (clinical trial NCT02319369), advanced solid tumors or lymphomas (clinical trial NCT01877382), and relapsed or refractory multiple myeloma (clinical trial NCT02579824) [104]. 

The HDM201 compound developed by Novartis is an imidazopyrrolidinone scaffold-based inhibitor of MDM2 which showed a significant anti-tumor activity in vitro and more efficient and better pharmacokinetic and pharmacodynamic profiles in in vivo models [104]. The phase I clinical trial NCT02143635 is currently evaluating the efficacy/safety profile of HDM201 in patients with advanced solid and haematological p53 wild type tumors.

Other MDM2 antagonists are currently under investigation in clinical trials. The phase I clinical study NCT00676910 is determining the safety and dosing of JNJ-26854165 in patients with advanced stage or refractory solid tumors [99]. At the preclinical stage, the novel MDM2 inhibitor MI-219 developed by Ascenta Therapeutics, designed to bind MDM2 in the p53-interaction sites has showed a potent and selective activity [99].

In addition, PXN727 and PXN822, which belong to a promising new class of inhibitors of the p53–MDM2 interactions, showed a strong efficacy and good safety profile in pre-clinical models [99]. 

A promising new therapeutic strategy to target the MDM2–p53 interaction is well exemplified by the mechanism of action of the compound RITA, identified by the National Cancer Institute. In fact, RITA which stands for reactivating P53 and inducing tumour apoptosis acts differently from the above described compounds, since RITA is able to directly bind p53 and prevent the MDM2 interaction. RITA has demonstrated the potential to inhibit cancer growth in several in vitro and in vivo models [99]. 

## 8. Concluding Remarks

In this review, we have discussed several nucleolar functions and their implications in human cancer. Particular attention has been given to evidence that both canonical and recently emergent non-canonical functions can be efficiently targeted using several therapeutic approaches. A wide plethora of strategies has been identified and developed so far, and the classification of currently available old and emerging therapeutic strategies has been proposed in this review. Therapeutic approaches selectively targeting the components of ribosome biogenesis core complex machinery and cancer-related signalling pathways involved in ribosome biogenesis regulation have been reported to be more efficient and less toxic than older chemotherapeutic strategies based on targeting rDNA. In particular, the RNA Pol I inhibitors constitute an emerging and promising class of nucleolar-targeting agents able to selectively inhibit ribosome biogenesis over-activation in cancer cells. The completion of several clinical trials will provide more information about their efficacy and safety in the treatment of human cancer. Another promising therapeutic approach is certainly the targeting of nucleolar stress pathway. The currently available strategies able to induce the p53-mediated nucleolar stress pathway have been largely discussed. We highlighted, in particular, the inhibitors of MDM2-p53 axis as the most promising and clinically relevant approach in inducing the nucleolar stress pathway.

In conclusion, several recent works have revealed the presence in the nucleolus of different proteins that, besides their role in ribosome biogenesis, are able to control different cellular processes and, in particular, are involved with different mechanisms in the control of cell proliferation. These studies have also highlighted the implication of the RNP-sequestering mechanism in several cancer processes. Understanding the molecular mechanism by which these proteins regulate morphology, biogenesis, and functions of the nucleolus, either canonical or non-canonical, will provide an important tool for the development of new targeted therapeutic approaches in cancer

## Figures and Tables

**Table 1:** Classification of Compounds targeting Nucleolar Components.

Drug	Class of Compounds	Mechanism of Action	Cancer Type
Doxorubicin	Anthracycline	rDNA intercalating agent/ topoisomerase II inhibitors	Haematological cancers, bladder, breast, stomach, lung, ovarian and thyroid cancer, sarcoma [29]
Cisplatin, Oxaliplatin	Platinum compound	rDNA crosslinking agent	Sarcoma, lymphoma, carcinoma [37,78,79]
Actinomycin D	Antibiotic	DNA intercalating agent	Wilms’ tumour, sarcoma [37]
Mitomycin C	Antibiotic	rDNA alkylating/crosslinking agent	Stomach or pancreatic adenocarcinoma; anal, bladder, breast, cervical, colorectal, head, neck, non-small-cell lung cancer [29]
Irinotecan/Topotecan	Camptothecins	Topoisomerase I inhibition	Ovarian, lung, cervical cancer [79]
Etoposide	Epipodophyllotoxins	Topoisomerase II inhibition	Sarcoma, glioblastoma, lung, testicular, haematological cancers [79]
5-flurouracil	Pyrimidine nucleotide analogue	Thymidylate synthase/rRNA/rDNA synthesis inhibitor	Colon, rectum, head, neck cancers [37,79,80]
Roscovitine/Olomoucine DRB	Cdk inhibitors	Disrupting nucleolar integrity	Adenocarcinoma, B-cell malignancies, breast cancer [81]
Flavopereirine (PB-100)	Alkaloid	Accumulating in the nucleoli	Glioblastoma [82]
Nanoparticles (SiO_2_)	Nanoparticles	Inducing nucleolar protein aggregates	ND [83]
Nanoparticles (TiO_2_)	Oligonucleotide-conjugated Nanoparticles	Depleting rDNA	ND [84]
Nanoparticles (Gold)	Nanoparticles	Interfering with the transcription of ribosomal DNA	Breast cancer [85]
DNA aptamers Naphthalene diimides	G-quadruplex interacting compounds	Binding to rDNA	Breast, lung cancer [86,87]

**Table 2:** Classification of Compounds selectively targeting Nucleolar Functions in Cancer.

Drug	Class of Compounds	Mechanism of Action	Cancer Type
CX-3543	Selective inhibitor of RNA Pol I	Targeting and disrupting nucleolin/rDNA G-quadruplex complexes	Carcinoid/neuroendocrine tumours [88]
CX-5461 9-Hydroxyellipticine (9HE) BMH-21	Selective inhibitor of RNA Pol I	Inhibiting RNA Pol I activity	Haematological cancers [32,89], breast cancer [90] Pre-clinical models [91,92,93,94,95]
Rapamycin Everolimus	mTOR signalling inhibitor	Inhibiting mTOR signalling resulting in inhibition of ribosome biogenesis	Renal cell carcinoma, breast cancer and lymphoma [96]
AKTi-1/2 MK-2206	AKT signalling inhibitor	Inhibiting AKT signalling resulting in suppression of rDNA gene transcription	Non–small cell lung cancer [97]
Nutlin-1/2/3, RG7112 RG7388 PXN727 PXN822	Nutlins and derivatives	Targeting the MDM2-P53 interaction	Haematological tumours, solid tumours [98,99]; osteosarcoma, head and neck cancer [100]; pre-clinical models [99]
MI-77301 MI-219	Spirooxindole-based compound	Targeting the MDM2-P53 interaction	Osteosarcoma, acute leukemia, prostate and colon cancer cells [101]; hematologic neoplasms and advanced solid tumors [99]
MK-8242	antimetabolite analogue of cytidine	Targeting the MDM2-P53 interaction	Solid tumors and haematological cancers [101]
AMG 232	Piperidinone derivative	Targeting the MDM2-P53 interaction	Breast cancer [102]
CGM097	dihydroisoquinolinone derivative	Targeting the MDM2-P53 interaction	Hematological and pediatric cancers [103]
DS3032b HDM201	Imidazopyrrolidinone derivative	Targeting the MDM2-P53 interaction	Haematological malignancies and advanced solid tumors [104]
JNJ-26854165	Tryptamine derivative	Targeting the MDM2-P53 interaction	Solid tumours [99]
RITA	Thiophen derivative	Targeting the MDM2-P53 interaction	Pre-clinical models [99]
p53-SLP	P53-synthetic long peptide vaccine	Stimulating immunoresponse against P53	Ovarian and colorectal cancer [99]

## References

[1] Pederson T. (2011). The nucleolus. Cold Spring Harb. Perspect. Biol..

[2] Tsekrekou M., Stratigi K., Chatzinikolaou G. (2017). The Nucleolus: In Genome Maintenance and Repair. Int. J. Mol. Sci..

[3] Nazar R.N. (2004). Ribosomal RNA processing and ribosome biogenesis in eukaryotes. IUBMB Life.

[4] Puvion-Dutilleul F., Puvion E., Bachellerie J.P. (1997). Early stages of pre-rRNA formation within the nucleolar ultrastructure of mouse cells studied by in situ hybridization with a 5’ETS leader probe. Chromosoma.

[5] Russell J., Zomerdijk J.C. (2006). The RNA polymerase I transcription machinery. Biochem. Soc. Symp..

[6] Grummt I. (2003). Life on a planet of its own: regulation of RNA polymerase I transcription in the nucleolus. Genes Dev..

[7] Russo G., Ricciardelli G., Pietropaolo C. (1997). Different domains cooperate to target the human ribosomal L7a protein to the nucleus and to the nucleoli. J. Biol. Chem..

[8] Andersen J.S., Lam Y.W., Leung A.K., Ong S.E., Lyon C.E., Lamond A.I., Mann M. (2005). Nucleolar proteome dynamics. Nature.

[9] Lindström M.S., Jurada D., Bursac S., Orsolic I., Bartek J., Volarevic S. (2018). Nucleolus as an emerging hub in maintenance of genome stability and cancer pathogenesis. Oncogene.

[10] Schöfer C., Weipoltshammer K. (2018). Nucleolus and chromatin. Histochem. Cell Biol..

[11] Tsai R.Y., Pederson T. (2014). Connecting the nucleolus to the cell cycle and human disease. FASEB J..

[12] Dillinger S., Straub T., Németh A. (2017). Nucleolus association of chromosomal domains is largely maintained in cellular senescence despite massive nuclear reorganisation. PLoS ONE.

[13] Russo A., Russo G. (2017). Ribosomal Proteins Control or Bypass p53 during Nucleolar Stress. Int. J. Mol. Sci..

[14] Golomb L., Volarevic S., Oren M. (2014). p53 and ribosome biogenesis stress: the essentials. FEBS Lett..

[15] Weeks S.E., Metge B.J., Samant R.S. (2019). The nucleolus: A central response hub for the stressors that drive cancer progression. Cell Mol. Life Sci..

[16] Iarovaia O.V., Minina E.P., Sheval E.V., Onichtchouk D., Dokudovskaya S., Razin S.V., Vassetzky Y.S. (2019). Nucleolus: A Central Hub for Nuclear Functions. Trends Cell Biol..

[17] Boisvert F.M., van Koningsbruggen S., Navascués J., Lamond A.I. (2007). The multifunctional nucleolus. Nat. Rev. Mol. Cell Biol..

[18] Woods S.J., Hannan K.M., Pearson R.B., Hannan R.D. (2015). The nucleolus as a fundamental regulator of the p53 response and a new target for cancer therapy. Biochim. Biophys. Acta.

[19] Derenzini M., Montanaro L., Treré D. (2009). What the nucleolus says to a tumour pathologist. Histopathology.

[20] Catez F., Dalla Venezia N., Marcel V., Zorbas C., Lafontaine D.L.J., Diaz J.J. (2019). Ribosome biogenesis: An emerging druggable pathway for cancer therapeutics. Biochem. Pharmacol..

[21] Derenzini M., Trerè D., Pession A., Montanaro L., Sirri V., Ochs R.L. (1998). Nucleolar function and size in cancer cells. Am. J. Pathol..

[22] Derenzini M., Nardi F., Farabegoli F., Ottinetti A., Roncaroli F., Bussolati G. (1989). Distribution of silver-stained interphase nucleolar organizer regions as a parameter to distinguish neoplastic from nonneoplastic reactive cells in human effusions. Acta Cytol..

[23] Montanaro L., Treré D., Derenzini M. (2008). Nucleolus, ribosomes, and cancer. Am. J. Pathol..

[24] Ruggero D. (2012). Revisiting the nucleolus: From marker to dynamic integrator of cancer signaling. Sci. Signal..

[25] van Sluis M., McStay B. (2014). Ribosome biogenesis: Achilles heel of cancer?. Genes Cancer.

[26] Boussemart L., Malka-Mahieu H., Girault I., Allard D., Hemmingsson O., Tomasic G., Thomas M., Basmadjian C., Ribeiro N., Thuaud F. (2014). eIF4F is a nexus of resistance to anti-BRAF and anti-MEK cancer therapies. Nature.

[27] Hsieh A.C., Costa M., Zollo O., Davis C., Feldman M.E., Testa J.R., Meyuhas O., Shokat K.M., Ruggero D. (2010). Genetic dissection of the oncogenic mTOR pathway reveals druggable addiction to translational control via 4EBP-eIF4E. Cancer Cell.

[28] Montanaro L., Treré D., Derenzini M. (2012). Changes in ribosome biogenesis may induce cancer by down-regulating the cell tumor suppressor potential. Biochim. Biophys. Acta.

[29] Bywater M.J., Pearson R.B., McArthur G.A., Hannan R.D. (2013). Dysregulation of the basal RNA polymerase transcription apparatus in cancer. Nat. Rev. Cancer.

[30] Uemura M., Zheng Q., Koh C.M., Nelson W.G., Yegnasubramanian S., De Marzo A.M. (2012). Overexpression of ribosomal RNA in prostate cancer is common but not linked to rDNA promoter hypomethylation. Oncogene.

[31] Williamson D., Lu Y.J., Fang C., Pritchard-Jones K., Shipley J. (2006). Nascent pre-rRNA overexpression correlates with an adverse prognosis in alveolar rhabdomyosarcoma. Genes Chromosomes Cancer.

[32] Bywater M.J., Poortinga G., Sanij E., Hein N., Peck A., Cullinane C., Wall M., Cluse L., Drygin D., Anderes K. (2012). Inhibition of RNA polymerase I as a therapeutic strategy to promote cancer-specific activation of p53. Cancer Cell.

[33] Drygin D., Rice W.G., Grummt I. (2010). The RNA polymerase I transcription machinery: an emerging target for the treatment of cancer. Annu. Rev. Pharmacol. Toxicol..

[34] Whittaker S., Martin M., Marais R. (2010). All roads lead to the ribosome. Cancer Cell.

[35] Tzoneva G., Perez-Garcia A., Carpenter Z., Khiabanian H., Tosello V., Allegretta M., Paietta E., Racevskis J., Rowe J.M., Tallman M.S. (2013). Activating mutations in the NT5C2 nucleotidase gene drive chemotherapy resistance in relapsed ALL. Nat. Med..

[36] De Keersmaecker K., Atak Z.K., Li N., Vicente C., Patchett S., Girardi T., Gianfelici V., Geerdens E., Clappier E., Porcu M. (2013). Exome sequencing identifies mutation in CNOT3 and ribosomal genes RPL5 and RPL10 in T-cell acute lymphoblastic leukemia. Nat. Genet..

[37] Hein N., Hannan K.M., George A.J., Sanij E., Hannan R.D. (2013). The nucleolus: An emerging target for cancer therapy. Trends Mol. Med..

[38] Peddibhotla S., Wei Z., Papineni R., Lam M.H., Rosen J.M., Zhang P. (2011). The DNA damage effector Chk1 kinase regulates Cdc14B nucleolar shuttling during cell cycle progression. Cell Cycle.

[39] Sasaki M., Kawahara K., Nishio M., Mimori K., Kogo R., Hamada K., Itoh B., Wang J., Komatsu Y., Yang Y.R. (2011). Regulation of the MDM2-P53 pathway and tumor growth by PICT1 via nucleolar RPL11. Nat. Med..

[40] Andrique L., Fauvin D., El Maassarani M., Colasson H., Vannier B., Séité P. (2012). ErbB3(80 kDa), a nuclear variant of the ErbB3 receptor, binds to the Cyclin D1 promoter to activate cell proliferation but is negatively controlled by p14ARF. Cell Signal..

[41] Audas T.E., Jacob M.D., Lee S. (2012). Immobilization of proteins in the nucleolus by ribosomal intergenic spacer noncoding RNA. Mol. Cell.

[42] Ebina M., Tsuruta F., Katoh M.C., Kigoshi Y., Someya A., Chiba T. (2013). Myeloma overexpressed 2 (Myeov2) regulates L11 subnuclear localization through Nedd8 modification. PLoS ONE.

[43] Qiu W., Wang G., Sun X., Ye J., Wei F., Shi X., Lv G. (2015). The involvement of cell surface nucleolin in the initiation of CCR6 signaling in human hepatocellular carcinoma. Med. Oncol..

[44] Daniely Y., Dimitrova D.D., Borowiec J.A. (2002). Stress-dependent nucleolin mobilization mediated by p53-nucleolin complex formation. Mol. Cell Biol..

[45] Kobayashi J., Fujimoto H., Sato J., Hayashi I., Burma S., Matsuura S., Chen D.J., Komatsu K. (2012). Nucleolin participates in DNA double-strand break-induced damage response through MDC1-dependent pathway. PLoS ONE.

[46] Ishimaru D., Zuraw L., Ramalingam S., Sengupta T.K., Bandyopadhyay S., Reuben A., Fernandes D.J., Spicer E.K. (2010). Mechanism of regulation of bcl-2 mRNA by nucleolin and A+U-rich element-binding factor 1 (AUF1). J. Biol. Chem..

[47] Lau A.W., Fukushima H., Wei W. (2012). The Fbw7 and betaTRCP E3 ubiquitin ligases and their roles in tumorigenesis. Front. Biosci. (Landmark Ed.).

[48] Boulon S., Westman B.J., Hutten S., Boisvert F.M., Lamond A.I. (2010). The nucleolus under stress. Mol. Cell.

[49] Fumagalli S., Ivanenkov V.V., Teng T., Thomas G. (2012). Suprainduction of p53 by disruption of 40S and 60S ribosome biogenesis leads to the activation of a novel G2/M checkpoint. Genes Dev..

[50] Zhang Y., Lu H. (2009). Signaling to p53: ribosomal proteins find their way. Cancer Cell.

[51] Di Matteo A., Franceschini M., Chiarella S., Rocchio S., Travaglini-Allocatelli C., Federici L. (2016). Molecules that target nucleophosmin for cancer treatment: an update. Oncotarget.

[52] Pfister A.S., Keil M., Kühl M. (2015). The Wnt Target Protein Peter Pan Defines a Novel p53-independent Nucleolar Stress-Response Pathway. J. Biol. Chem..

[53] Ayrault O., Andrique L., Fauvin D., Eymin B., Gazzeri S., Séité P. (2006). Human tumor suppressor p14ARF negatively regulates rRNA transcription and inhibits UBF1 transcription factor phosphorylation. Oncogene.

[54] Gjerset R.A., Bandyopadhyay K. (2006). Regulation of p14ARF through subnuclear compartmentalization. Cell Cycle.

[55] Russo A., Esposito D., Catillo M., Pietropaolo C., Crescenzi E., Russo G. (2013). Human rpL3 induces G₁/S arrest or apoptosis by modulating p21 (waf1/cip1) levels in a p53-independent manner. Cell Cycle.

[56] Esposito D., Crescenzi E., Sagar V., Loreni F., Russo A., Russo G. (2014). Human rpL3 plays a crucial role in cell response to nucleolar stress induced by 5-FU and L-OHP. Oncotarget.

[57] Russo G., Cuccurese M., Monti G., Russo A., Amoresano A., Pucci P., Pietropaolo C. (2005). Ribosomal protein L7a binds RNA through two distinct RNA-binding domains. Biochem. J..

[58] Russo A., Russo G., Cuccurese M., Garbi C., Pietropaolo C. (2006). The 3’-untranslated region directs ribosomal protein-encoding mRNAs to specific cytoplasmic regions. Biochim. Biophys. Acta.

[59] Russo A., Cirulli C., Amoresano A., Pucci P., Pietropaolo C., Russo G. (2008). cis-acting sequences and trans-acting factors in the localization of mRNA for mitochondrial ribosomal proteins. Biochim. Biophys. Acta.

[60] Cuccurese M., Russo G., Russo A., Pietropaolo C. (2005). Alternative splicing and nonsense-mediated mRNA decay regulate mammalian ribosomal gene expression. Nucleic Acids Res..

[61] Russo A., Siciliano G., Catillo M., Giangrande C., Amoresano A., Pucci P., Pietropaolo C., Russo G. (2010). hnRNP H1 and intronic G runs in the splicing control of the human rpL3 gene. Biochim. Biophys. Acta.

[62] Russo A., Catillo M., Esposito D., Briata P., Pietropaolo C., Russo G. (2011). Autoregulatory circuit of human rpL3 expression requires hnRNP H1, NPM and KHSRP. Nucleic Acids Res..

[63] d’Emmanuele di Villa Bianca R., Mitidieri E., Fusco F., Russo A., Pagliara V., Tramontano T., Donnarumma E., Mirone V., Cirino G., Russo G. (2016). Urothelium muscarinic activation phosphorylates CBS(Ser227) via cGMP/PKG pathway causing human bladder relaxation through H2S production. Sci. Rep..

[64] Russo A., Saide A., Cagliani R., Cantile M., Botti G., Russo G. (2016). rpL3 promotes the apoptosis of p53 mutated lung cancer cells by down-regulating CBS and NFκB upon 5-FU treatment. Sci. Rep..

[65] Pagliara V., Saide A., Mitidieri E., d’Emmanuele di Villa Bianca R., Sorrentino R., Russo G., Russo A. (2016). 5-FU targets rpL3 to induce mitochondrial apoptosis via cystathionine-β-synthase in colon cancer cells lacking p53. Oncotarget.

[66] De Filippis D., Russo A., D’Amico A., Esposito G., Pietropaolo C., Concetta P., Cinelli M., Russo G., Iuvone T. (2008). Cannabinoids reduce granuloma-associated angiogenesis in rats by controlling transcription and expression of mast cell protease-5. Br. J. Pharmacol..

[67] De Filippis D., Russo A., De Stefano D., Cipriano M., Esposito D., Grassia G., Carnuccio R., Russo G., Iuvone T. (2014). Palmitoylethanolamide inhibits rMCP-5 expression by regulating MITF activation in rat chronic granulomatous inflammation. Eur. J. Pharmacol..

[68] Russo A., Pagliara V., Albano F., Esposito D., Sagar V., Loreni F., Irace C., Santamaria R., Russo G. (2016). Regulatory role of rpL3 in cell response to nucleolar stress induced by Act D in tumor cells lacking functional p53. Cell Cycle.

[69] Russo A., Pellosi D.S., Pagliara V., Milone M.R., Pucci B., Caetano W., Hioka N., Budillon A., Ungaro F., Russo G. (2016). Biotin-targeted Pluronic(^®^) P123/F127 mixed micelles delivering niclosamide: A repositioning strategy to treat drug-resistant lung cancer cells. Int. J. Pharm..

[70] Russo A., Saide A., Smaldone S., Faraonio R., Russo G. (2017). Role of uL3 in Multidrug Resistance in p53-Mutated Lung Cancer Cells. Int. J. Mol. Sci..

[71] Russo A., Maiolino S., Pagliara V., Ungaro F., Tatangelo F., Leone A., Scalia G., Budillon A., Quaglia F., Russo G. (2016). Enhancement of 5-FU sensitivity by the proapoptotic rpL3 gene in p53 null colon cancer cells through combined polymer nanoparticles. Oncotarget.

[72] Yoshikawa M., Fujii Y.R. (2016). Human Ribosomal RNA-Derived Resident MicroRNAs as the Transmitter of Information upon the Cytoplasmic Cancer Stress. Biomed. Res. Int..

[73] Zheng D., Zhang J., Ni J., Luo J., Wang J., Tang L., Zhang L., Wang L., Xu J., Su B. (2015). Small nucleolar RNA 78 promotes the tumorigenesis in non-small cell lung cancer. J. Exp. Clin. Cancer Res..

[74] Carotenuto P., Fassan M., Pandolfo R., Lampis A., Vicentini C., Cascione L., Paulus-Hock V., Boulter L., Guest R., Quagliata L. (2017). Wnt signalling modulates transcribed-ultraconserved regions in hepatobiliary cancers. Gut.

[75] Pirogov S.A., Gvozdev V.A., Klenov M.S. (2019). Long Noncoding RNAs and Stress Response in the Nucleolus. Cells.

[76] Xu B., Li H., Perry J.M., Singh V.P., Unruh J., Yu Z., Zakari M., McDowell W., Li L., Gerton J.L. (2017). Ribosomal DNA copy number loss and sequence variation in cancer. PLoS Genet..

[77] Quin J.E., Devlin J.R., Cameron D., Hannan K.M., Pearson R.B., Hannan R.D. (2014). Targeting the nucleolus for cancer intervention. Biochim. Biophys. Acta.

[78] Cheung-Ong K., Giaever G., Nislow C. (2013). DNA-damaging agents in cancer chemotherapy: Serendipity and chemical biology. Chem. Biol..

[79] Burger K., Mühl B., Harasim T., Rohrmoser M., Malamoussi A., Orban M., Kellner M., Gruber-Eber A., Kremmer E., Hölzel M. (2010). Chemotherapeutic drugs inhibit ribosome biogenesis at various levels. J. Biol. Chem..

[80] Sun X.X., Dai M.S., Lu H. (2007). 5-fluorouracil activation of p53 involves an MDM2-ribosomal protein interaction. J. Biol. Chem..

[81] Burger K., Mühl B., Rohrmoser M., Coordes B., Heidemann M., Kellner M., Gruber-Eber A., Heissmeyer V., Strässer K., Eick D. (2013). Cyclin-dependent kinase 9 links RNA polymerase II transcription to processing of ribosomal RNA. J. Biol. Chem..

[82] Beljanski M., Crochet S. (1995). The anticancer agent pb-100 concentrates in the nucleus and nucleoli of human glioblastoma cells but does not enter normal astrocytes. Int. J. Oncol..

[83] Chen M., von Mikecz A. (2005). Formation of nucleoplasmic protein aggregates impairs nuclear function in response to SiO2 nanoparticles. Exp. Cell Res..

[84] Paunesku T., Vogt S., Lai B., Maser J., Stojićević N., Thurn K.T., Osipo C., Liu H., Legnini D., Wang Z. (2007). Intracellular distribution of TiO2-DNA oligonucleotide nanoconjugates directed to nucleolus and mitochondria indicates sequence specificity. Nano Lett..

[85] Kodiha M., Mahboubi H., Maysinger D., Stochaj U. (2016). Gold Nanoparticles Impinge on Nucleoli and the Stress Response in MCF7 Breast Cancer Cells. Nanobiomedicine (Rij).

[86] Esposito V., Russo A., Vellecco V., Bucci M., Russo G., Mayol L., Virgilio A., Galeone A. (2018). Thrombin binding aptamer analogues containing inversion of polarity sites endowed with antiproliferative and anti-motility properties against Calu-6 cells. Biochim. Biophys. Acta Gen. Subj..

[87] Pirota V., Nadai M., Doria F., Richter S.N. (2019). Naphthalene Diimides as Multimodal G-Quadruplex-Selective Ligands. Molecules.

[88] Drygin D., Siddiqui-Jain A., O’Brien S., Schwaebe M., Lin A., Bliesath J., Ho C.B., Proffitt C., Trent K., Whitten J.P. (2009). Anticancer activity of CX-3543: A direct inhibitor of rRNA biogenesis. Cancer Res..

[89] Drygin D., Lin A., Bliesath J., Ho C.B., O’Brien S.E., Proffitt C., Omori M., Haddach M., Schwaebe M.K., Siddiqui-Jain A. (2011). Targeting RNA polymerase I with an oral small molecule CX-5461 inhibits ribosomal RNA synthesis and solid tumor growth. Cancer Res..

[90] Andrews W.J., Panova T., Normand C., Gadal O., Tikhonova I.G., Panov K.I. (2013). Old drug, new target: ellipticines selectively inhibit RNA polymerase I transcription. J. Biol. Chem..

[91] Peltonen K., Colis L., Liu H., Jäämaa S., Zhang Z., Af Hällström T., Moore H.M., Sirajuddin P., Laiho M. (2014). Small molecule BMH-compounds that inhibit RNA polymerase I and cause nucleolar stress. Mol. Cancer Ther..

[92] Colis L., Peltonen K., Sirajuddin P., Liu H., Sanders S., Ernst G., Barrow J.C., Laiho M. (2014). DNA intercalator BMH-21 inhibits RNA polymerase I independent of DNA damage response. Oncotarget.

[93] Peltonen K., Colis L., Liu H., Trivedi R., Moubarek M.S., Moore H.M., Bai B., Rudek M.A., Bieberich C.J., Laiho M. (2014). A targeting modality for destruction of RNA polymerase I that possesses anticancer activity. Cancer Cell.

[94] Wei T., Najmi S.M., Liu H., Peltonen K., Kucerova A., Schneider D.A., Laiho M. (2018). Small-Molecule Targeting of RNA Polymerase I Activates a Conserved Transcription Elongation Checkpoint. Cell Rep..

[95] Fu X., Xu L., Qi L., Tian H., Yi D., Yu Y., Liu S., Li S., Xu Y., Wang C. (2017). BMH-21 inhibits viability and induces apoptosis by p53-dependent nucleolar stress responses in SKOV3 ovarian cancer cells. Oncol. Rep..

[96] Wall M., Poortinga G., Stanley K.L., Lindemann R.K., Bots M., Chan C.J., Bywater M.J., Kinross K.M., Astle M.V., Waldeck K. (2013). The mTORC1 inhibitor everolimus prevents and treats Eμ-Myc lymphoma by restoring oncogene-induced senescence. Cancer Discov..

[97] Chan J.C., Hannan K.M., Riddell K., Ng P.Y., Peck A., Lee R.S., Hung S., Astle M.V., Bywater M., Wall M. (2011). AKT promotes rRNA synthesis and cooperates with c-MYC to stimulate ribosome biogenesis in cancer. Sci. Signal..

[98] Andreeff M., Kelly K.R., Yee K., Assouline S., Strair R., Popplewell L., Bowen D., Martinelli G., Drummond M.W., Vyas P. (2016). Results of the Phase I Trial of RG7112, a Small-Molecule MDM2 Antagonist in Leukemia. Clin. Cancer Res..

[99] Cheok C.F., Verma C.S., Baselga J., Lane D.P. (2011). Translating p53 into the clinic. Nat. Rev. Clin. Oncol..

[100] Ding Q., Zhang Z., Liu J.J., Jiang N., Zhang J., Ross T.M., Chu X.J., Bartkovitz D., Podlaski F., Janson C. (2013). Discovery of RG7388, a potent and selective p53-MDM2 inhibitor in clinical development. J. Med. Chem..

[101] Wang S., Zhao Y., Aguilar A., Bernard D., Yang C.Y. (2017). Targeting the MDM2-p53 Protein-Protein Interaction for New Cancer Therapy: Progress and Challenges. Cold Spring Harb. Perspect. Med..

[102] Canon J., Osgood T., Olson S.H., Saiki A.Y., Robertson R., Yu D., Eksterowicz J., Ye Q., Jin L., Chen A. (2015). The MDM2 Inhibitor AMG 232 Demonstrates Robust Antitumor Efficacy and Potentiates the Activity of p53-Inducing Cytotoxic Agents. Mol. Cancer Ther..

[103] Holzer P., Masuya K., Furet P., Kallen J., Valat-Stachyra T., Ferretti S., Berghausen J., Bouisset-Leonard M., Buschmann N., Pissot-Soldermann C. (2015). Discovery of a Dihydroisoquinolinone Derivative (NVP-CGM097): A Highly Potent and Selective MDM2 Inhibitor Undergoing Phase 1 Clinical Trials in p53wt Tumors. J. Med. Chem..

[104] Tisato V., Voltan R., Gonelli A., Secchiero P., Zauli G. (2017). MDM2/X inhibitors under clinical evaluation: perspectives for the management of hematological malignancies and pediatric cancer. J. Hematol. Oncol..

[105] Ratnadiwakara M., Änkö M. (2018). mRNA Stability Assay Using Transcription Inhibition by Actinomycin D in Mouse Pluripotent Stem Cells. Bio-protocol.

[106] Latonen L., Moore H.M., Bai B., Jäämaa S., Laiho M. (2011). Proteasome inhibitors induce nucleolar aggregation of proteasome target proteins and polyadenylated RNA by altering ubiquitin availability. Oncogene.

[107] Navon A., Ciechanover A. (2009). The 26 S proteasome: From basic mechanisms to drug targeting. J. Biol. Chem..

[108] Galimberti V., Kinor N., Shav-Tal Y., Biggiogera M., Brüning A. (2016). The stress-inducible transcription factor ATF4 accumulates at specific rRNA-processing nucleolar regions after proteasome inhibition. Eur. J. Cell Biol..

[109] Maiolino S., Russo A., Pagliara V., Conte C., Ungaro F., Russo G., Quaglia F. (2015). Biodegradable nanoparticles sequentially decorated with Polyethyleneimine and Hyaluronan for the targeted delivery of docetaxel to airway cancer cells. J. Nanobiotechnol..

[110] d’Angelo I., Costabile G., Durantie E., Brocca P., Rondelli V., Russo A., Russo G., Miro A., Quaglia F., Petri-Fink A. (2018). Hybrid Lipid/Polymer Nanoparticles for Pulmonary Delivery of siRNA: Development and Fate Upon In Vitro Deposition on the Human Epithelial Airway Barrier. J. Aerosol. Med. Pulm. Drug Deliv..

[111] Esposito V., Russo A., Amato T., Varra M., Vellecco V., Bucci M., Russo G., Virgilio A., Galeone A. (2017). Backbone modified TBA analogues endowed with antiproliferative activity. Biochim. Biophys. Acta Gen. Subj..

[112] Virgilio A., Russo A., Amato T., Russo G., Mayol L., Esposito V., Galeone A. (2017). Monomolecular G-quadruplex structures with inversion of polarity sites: new topologies and potentiality. Nucleic Acids Res..

[113] Esposito V., Russo A., Amato T., Vellecco V., Bucci M., Mayol L., Russo G., Virgilio A., Galeone A. (2018). The “Janus face” of the thrombin binding aptamer: Investigating the anticoagulant and antiproliferative properties through straightforward chemical modifications. Bioorg Chem..

[114] Xu H., Di Antonio M., McKinney S., Mathew V., Ho B., O’Neil N.J., Santos N.D., Silvester J., Wei V., Garcia J. (2017). CX-5461 is a DNA G-quadruplex stabilizer with selective lethality in BRCA1/2 deficient tumours. Nat. Commun..

[115] Drygin D., O’Brien S.E., Hannan R.D., McArthur G.A., Von Hoff D.D. (2014). Targeting the nucleolus for cancer-specific activation of p53. Drug Discov. Today.

[116] Peltonen K., Colis L., Liu H., Jäämaa S., Moore H.M., Enbäck J., Laakkonen P., Vaahtokari A., Jones R.J., af Hällström T.M. (2010). Identification of novel p53 pathway activating small-molecule compounds reveals unexpected similarities with known therapeutic agents. PLoS ONE.

[117] Voit R., Hoffmann M., Grummt I. (1999). Phosphorylation by G1-specific cdk-cyclin complexes activates the nucleolar transcription factor UBF. EMBO J..

[118] Zhai W., Comai L. (2000). Repression of RNA polymerase I transcription by the tumor suppressor p53. Mol. Cell Biol..

[119] Kruhlak M., Crouch E.E., Orlov M., Montaño C., Gorski S.A., Nussenzweig A., Misteli T., Phair R.D., Casellas R. (2007). The ATM repair pathway inhibits RNA polymerase I transcription in response to chromosome breaks. Nature.

[120] Ma H., Pederson T. (2013). The nucleolus stress response is coupled to an ATR-Chk1-mediated G2 arrest. Mol. Biol. Cell.

[121] Calkins A.S., Iglehart J.D., Lazaro J.B. (2013). DNA damage-induced inhibition of rRNA synthesis by DNA-PK and PARP-1. Nucleic Acids Res..

[122] Bierhoff H., Dundr M., Michels A.A., Grummt I. (2008). Phosphorylation by casein kinase 2 facilitates rRNA gene transcription by promoting dissociation of TIF-IA from elongating RNA polymerase I. Mol. Cell Biol..

[123] Bakshi R., Zaidi S.K., Pande S., Hassan M.Q., Young D.W., Montecino M., Lian J.B., van Wijnen A.J., Stein J.L., Stein G.S. (2008). The leukemogenic t(8;21) fusion protein AML1-ETO controls rRNA genes and associates with nucleolar-organizing regions at mitotic chromosomes. J. Cell Sci..

[124] Anglin I., Passaniti A. (2004). Runx protein signaling in human cancers. Cancer Treat. Res..

[125] Fukawa T., Ono M., Matsuo T., Uehara H., Miki T., Nakamura Y., Kanayama H.O., Katagiri T. (2012). DDX31 regulates the p53-HDM2 pathway and rRNA gene transcription through its interaction with NPM1 in renal cell carcinomas. Cancer Res..

[126] Delloye-Bourgeois C., Goldschneider D., Paradisi A., Therizols G., Belin S., Hacot S., Rosa-Calatrava M., Scoazec J.Y., Diaz J.J., Bernet A. (2012). Nucleolar localization of a netrin-1 isoform enhances tumor cell proliferation. Sci. Signal..

[127] Zhang Y., Forys J.T., Miceli A.P., Gwinn A.S., Weber J.D. (2011). Identification of DHX33 as a mediator of rRNA synthesis and cell growth. Mol. Cell Biol..

[128] Cheng Y., Liang P., Geng H., Wang Z., Li L., Cheng S.H., Ying J., Su X., Ng K.M., Ng M.H. (2012). A novel 19q13 nucleolar zinc finger protein suppresses tumor cell growth through inhibiting ribosome biogenesis and inducing apoptosis but is frequently silenced in multiple carcinomas. Mol. Cancer Res..

[129] Hannan K.M., Brandenburger Y., Jenkins A., Sharkey K., Cavanaugh A., Rothblum L., Moss T., Poortinga G., McArthur G.A., Pearson R.B. (2003). mTOR-dependent regulation of ribosomal gene transcription requires S6K1 and is mediated by phosphorylation of the carboxy-terminal activation domain of the nucleolar transcription factor UBF. Mol. Cell Biol..

[130] Brown C.J., Cheok C.F., Verma C.S., Lane D.P. (2011). Reactivation of p53: from peptides to small molecules. Trends Pharmacol. Sci..

[131] Inoue K., Fry E.A. (2016). Aberrant splicing of the DMP1-ARF-MDM2-p53 pathway in cancer. Int. J. Cancer.

[132] Patnaik A., Tolcher A., Beeram M., Nemunaitis J., Weiss G.J., Bhalla K., Agrawal M., Nichols G., Middleton S., Beryozkina A. (2015). Clinical pharmacology characterization of RG7112, an MDM2 antagonist, in patients with advanced solid tumors. Cancer Chemother. Pharmacol..

